# Sedentary behaviour is associated with increased long-term cardiovascular risk in patients with rheumatoid arthritis independently of moderate-to-vigorous physical activity

**DOI:** 10.1186/s12891-017-1473-9

**Published:** 2017-03-29

**Authors:** Sally A. M. Fenton, Jet J. C. S. Veldhuijzen van Zanten, George D. Kitas, Joan L. Duda, Peter C. Rouse, Chen-an Yu, George S. Metsios

**Affiliations:** 1 School of Sport, Exercise and Rehabilitation Sciences, University of Birmingham, Birmingham, UK; 2 Faculty of Health Education and Wellbeing, University of Wolverhampton, West Midlands, UK; 3 Department of Rheumatology, Russells Hall Hospital, Dudley Group NHS Foundation Trust, Dudley, UK; 4 Department for Health, University of Bath, Bath, UK; 5 Faculty of Biological Sciences, University of Leeds, Leeds, UK

**Keywords:** Sedentary behaviour, Physical activity, Rheumatoid Arthritis, Accelerometry, Cardiovascular risk, Inflammation, Physical Function

## Abstract

**Background:**

Rheumatoid Arthritis (RA) is associated with an increased risk of cardiovascular disease (CVD). The physical dysfunction symptomatic of RA means people living with this disease spend large periods of the day sedentary, which may further elevate their risk of CVD. The primary aim of this study was to investigate relationships between objectively assessed sedentary behaviour patterns and light physical activity (LPA) with 10-year risk of CVD. Secondary aims were to explore the role of sedentary behaviour patterns and LPA for individual CVD risk factors and functional disability in RA. The extent to which associations were independent of moderate-to-vigorous physical activity (MVPA) engagement was also examined.

**Methods:**

Baseline data from a subsample of participants recruited to the Physical Activity in Rheumatoid Arthritis (PARA) study were used to answer current research questions. Sixty-one patients with RA (mean age (± SD) = 54.92 ± 12.39 years) provided a fasted blood sample and underwent physical assessments to evaluate factors associated with their cardiovascular health. Sedentary behaviour patterns (sedentary time, sedentary bouts, sedentary breaks), LPA and MVPA were measured via 7-days of accelerometry. Ten-year CVD risk was computed (Q-risk-score2), and functional disability determined via questionnaire.

**Results:**

Regressions revealed significant positive associations between sedentary time and the number of sedentary bouts per day ≥20 min with 10-year CVD risk, with the reverse true for LPA participation. Associations were independent of MVPA engagement.

**Conclusions:**

Promoting LPA participation and restricting sedentary bouts to <20 min may attenuate long-term CVD risk in RA, independent of MVPA engagement.

**Trial registration:**

ISRCTN04121489 (retrospectively registered 19/10/2012).

## Background

Rheumatoid Arthritis (RA) is a chronic, autoimmune inflammatory disease affecting 0.5–1% of the population, manifesting with joint swelling, cartilage destruction and bone erosion. These clinical characteristics contribute towards joint pain and impaired physical function symptomatic of the disease [1–3]. The heightened inflammatory-load seen in RA may also contribute to the increased risk of cardiovascular disease (CVD) and associated morbidity and mortality [4–10].

It is well evidenced that higher levels of physical activity (PA) associate with improvement in cardiovascular health, systemic inflammation and physical function in RA [11–20]. This relationship is reported to occur in a ‘dose-response’ manner; with moderate-to-vigorous intensity physical activity [MVPA, i.e., ≥ 3 metabolic equivalents (METS)] demonstrated to be particularly beneficial for RA outcomes [16–20]. However, recent studies conducted among older adults and non-RA clinical populations, have underlined the role of engagement in light intensity physical activity (LPA, i.e., 1.6 – 2.9 METS) for reducing cardiovascular risk, improving metabolic health and attenuating functional disability [21–28]. Thus, engagement in LPA may also be relevant to improving pertinent RA outcomes.

Still, despite evidence highlighting the positive consequences of PA engagement for RA outcomes, people living with RA remain physically inactive, and are often referred to as “sedentary” [29–31]. However, it is important to recognise that whilst both sedentary behaviour and physical inactivity represent the ‘non-exercise’ part of the physical activity continuum, they are distinct constructs, and can be operationalised as such [11, 32, 33]. Specifically, physically inactivity refers to failure to meet the recommended 150 min of MVPA per week [33, 34], where sedentary behaviour is defined as any waking behaviour resulting in energy expenditure ≤ 1.5 METS whilst sitting or lying [32, 35].

Sedentary behaviour is demonstrated to be adversely linked to several health outcomes which are relevant to RA. For example, studies have revealed time spent sedentary to hold deleterious consequences for cardiovascular and cardio-metabolic health, as well as functional ability for both healthy adults and patient groups [21, 36–40]. Moreover, the way sedentary time is accumulated is reported to hold implications for CVD risk and physical function. Specifically, the number and length of uninterrupted sedentary periods (i.e., sedentary bouts), and the frequency of interruptions in sedentary time with standing and﻿/or LPA (i.e., sedentary breaks) are reported to be associated with markers of cardio-metabolic and cardiovascular health, and physical dysfunction in older cohorts [36, 37, 41–48]. However, research is yet to examine the implications of objectively measured sedentary behaviour patterns and LPA for risk of CVD (and other relevant health outcomes) in RA [49].

The primary aim of this study was therefore to examine the relationships between objectively assessed sedentary time, sedentary behaviour patterns (bouts and breaks) and LPA with global long-term (10-year) CVD risk among people living with RA. The extent to which associations are independent of levels of MVPA participation will also be examined. Indeed, interventions which focus on reducing sedentary behaviour and increasing LPA (i.e., promoting the “sit less, move more” message), will likely only be efficacious towards reducing risk of CVD in the instance these behaviours are favourably associated with CVD ﻿risk/profile, independently of the possible cardio-protective effects of MVPA engagement. We hypothesise that higher levels of daily sedentary time, and more frequent (and longer) uninterrupted sedentary bouts, will be adversely associated with 10-year CVD risk, after considering daily levels of MVPA participation. In contrast, we expect that the number of sedentary breaks per day, and higher levels of engagement in LPA, will be beneficially associated with 10-year CVD risk, independently of MVPA engagement. Finally, given the lack of research examining the role of sedentary behaviour patterns and LPA for RA outcomes, secondary aims of this study were to explore the role of sedentary time, sedentary behaviour patterns and LPA with individual CVD risk factors and functional disability in RA.

## Methods

### Participants

Participants were patients recruited to the Physical Activity in Rheumatoid Arthritis (PARA) study (Trial Number: ISRCTN04121489), a randomised controlled trial with the primary aim of improving cardiorespiratory fitness among RA patients [50]. All participants fulfilled diagnostic criteria for RA as outlined by the American College of Rheumatology [51]. Exclusion criteria to the PARA study were recent joint surgery (in preceding 6 months), fibromyalgia and co-morbidity incompatible with exercise as per the ACSM guidelines. This study utilised baseline data collected during the PARA study. The study was approved by the local NHS Research Ethics Committee.

### Recruitment and protocol

Patients attending Rheumatology outpatient clinics at Russells Hall Hospital (Dudley, England), were provided with study information sheets. Interested participants (*N* = 97, mean age (± SD) = 58.7 ± 10.18 years) provided informed consent and attended two appointments one week apart. During the first visit, participants provided a fasted blood sample and were fitted with an accelerometer to wear for the next seven days. Participants attended their second visit one week later to return accelerometers, and undergo assessments to evaluate factors associated with their physical and cardiovascular health (e.g., height and weight [body-mass-index], blood pressure, self-reported functional disability).

### Measures

#### Objectively assessed sedentary behaviour patterns, LPA and MVPA

Sedentary behaviour patterns, LPA and MVPA were assessed using GT3X accelerometers (Actigraph) [52]. The GT3X detects movements over pre-specified time periods called epochs. Movement counts within each epoch are summed and converted to activity counts that are interpreted to determine frequency, intensity and duration of PA and sedentary time engagement. Accelerometers were initialised to measure PA in 60 s epochs. Participants wore the accelerometer for seven days, on the right hip, removing only for water-based activities (e.g., swimming and bathing).

##### Accelerometer data reduction

Data were downloaded from the GT3X and analysed using the Actilife software (Actilife version 6.2). Time filters were applied to the software, enabling extraction of data pertaining only to waking hours (i.e., 7:00 am – 10:30 pm). Data were cleaned and checked for spurious values and periods of non-wear. Non-wear time was determined by identifying strings of consecutive zeros recorded by the accelerometer for >60 min, allowing for 2 min of counts <100 [53]. Valid wear criteria was set as ≥10 h wear during waking hours on ≥4 days, including a weekend day [53, 54]. Participants meeting minimum accelerometer wear criteria (*N* = 61), were retained for inclusion in subsequent analyses (excluded, *N* = 36).

##### Computation of sedentary behaviour variables, LPA and MVPA

*Sedentary time, LPA* and *MVPA* were defined as <100, 100 – 2019, and ≥2020 counts per minute, respectively [53]. The counts-based method used to quantify time spent in these behaviours is derived from validation studies employing definitions of ≤1.5 (sedentary behaviour), 1.6 – 2.9 (LPA) and ≥3 METs (MVPA) [53]. *Sedentary breaks* were calculated as interruptions in sedentary time (i.e., ≥100 counts per minute) for ≥1 min [41]. *Sedentary bout* variables were derived by determining the number and length of consecutive zeros recorded by the accelerometer, relative to a ≥20 min bout length [41]. Independent variables computed for statistical analysis were sedentary time (min/day), the average number of sedentary bouts ≥20 min (﻿number/day), the average time per sedentary bout ≥20 min (minutes), the number of sedentary breaks (number/﻿day)﻿, LPA (min/day) and MVPA (min/day).

### 10-year risk of CVD

Global 10-year CVD risk was determined via the calculation of the Q-risk score (Qrisk-2) [55]. Specifically, age, gender, ethnicity, physical assessments (height, weight, systolic blood pressure) total cholesterol/HDL ratio, self-reported smoking status, diabetic status, presence of kidney disease and family history of heart disease were used to determine participants Q-risk-score [55]. Height was measured to the nearest 0.5 cm using a standard height measure (Seca 214 Road Rod), and weight was determined using digital scales (Tanita Corporation). Blood pressure was assessed using an electronic sphygmomanometer (Datascope Accutor) using procedures previously described [56]. Total cholesterol and high density lipoprotein-cholesterol (HDL-C) were determined from venous blood samples collected in the fasting state (≥12 h fast), and analysed using routine laboratory procedures.

### Individual CVD risk factors

Individual CVD risk factors determined from fasted blood samples were; plasma markers of inflammation (C-reactive protein (CRP), erythrocyte sedimentation rate (ESR) and fibrinogen – measured using standard laboratory procedures or ELISAs), plasma lipids (total cholesterol, HDL-C, low density lipoprotein cholesterol (LDL-C) and triglycerides), blood pressure (systolic and diastolic) and plasma glucose and insulin. Homeostasis model assessment (HOMA) was also calculated to determine participants’ degree of insulin resistance.

### Functional disability

The Stanford Health Assessment Questionnaire (HAQ) was used to determine participants’ degree of functional disability [57]. The HAQ is comprised of 8 sections pertaining to different activities; *dressing, arising, eating, walking, hygiene, reach, grip, and activities*, Following the stem, “Are you able to….”, each section includes 2 or 3 questions (e.g., *dressing*, ‘shampoo your hair’), scored from 0 (without any difficulty) to 3 (unable to do). The score given to each section is the highest score reported within that section. The score is computed as the average of the sum of the scores from all 8 sections.

### Statistical analyses

Data were checked for normality using the Kolmogorov-Smirnov tests of normality; non-normally distributed data were log transformed (Table 1) and used in subsequent parametric statistical tests. Chi-square tests and one way analyses of variance (ANOVA) were conducted to determine the occurrence of sample bias related to participant exclusion on the basis of missing accelerometer data. For included participants, descriptive statistics were calculated and one way ANOVAs conducted to determine the presence of gender differences for all measured variables. In addition, one-way ANOVAs were carried out to examine significant differences in targeted outcomes based on current treatment (i.e., Disease Modifying Anti-Rheumatic Drugs (DMARDS), anti-TNF therapy, Non-Steroidal Anti-Inflammatory Drugs (NSAIDs), analgesics, cholesterol lowering medication, medication for hypertension). Subsequently, bivariate correlations between targeted variables were computed.

**Table 1:** Descriptive statistics for the total sample and sample stratified by gender

	Male (*N* = 20)	Female (*N* = 41)	Total (*N* = 61)
Age (years)	58.85 ± 9.44	53.00 ± 13.28	54.92 ± 12.39
Height (m)	1.73 ± 0.10	1.63 ± 0.06	1.66 ± 0.09
Weight (kg)	78.13 ± 10.33	76.81 ± 19.42	77.23 ± 16.94
Ethnicity (% Caucasian)	31.1	54.1	85.2
RA duration (years from diagnosis)	5.05 ± 5.44	7.97 ± 10.34	6.96 ± 9.01
Accelerometer data			
Average valid wear time (hours/day)	12.99 ± 0.74	13.12 ± 0.77	13.08 ± 0.76
Sedentary time (min/day)	505.40 ± 71.45	493.52 ± 67.24	497.42 ± 68.28
^a^ LPA (min/day)	251.14 ± 71.79	278.23 ± 67.22	269.35 ± 69.35
^a^ MVPA (min/day)	22.65 ± 22.53	15.70 ± 13.77	17.98 ± 17.26
Sedentary behaviour patterns			
Number of Sbreaks (average/day)	79.59 ± 16.89	86.52 ± 11.94	84.32 ± 14.02
Number of Sbouts ≥ 20 min (average/day)	6.37 ± 2.41	5.45 ± 1.91	5.75 ± 2.11
Average time per Sbouts ≥ 20 (min)	31.08 ± 2.07	30.64 ± 2.75	30.78 ± 2.54
10 year risk of CVD			
Q-risk (% risk of 10 year CVD)	24.14 ± 14.82	12.54 ± 11.05	16.33 ± 13.45^**^
Self-reported smoking status (% smokers)	3.65	3.65	7.3
Diabetic status (% diabetic)	1.8	5.5	7.3
Presence of kidney disease (% yes)	0	0	0
Family history of heart disease (% yes)	118.1	36.4	54.5
Individual CVD risk factors			
^a^ CRP (mg/L)	6.72 ± 8.12	7.66 ± 9.58	7.35 ± 9.09
^a^ ESR (mm/h)	10.00 ± 8.12	16.55 ± 16.50	14.37 ± 14.52
Fibrinogen (g/L)	4.64 ± 0.90	4.60 ± 1.20	4.62 ± 1.10
Total cholesterol (mmol/L)	4.77 ± 0.70	5.01 ± 1.09	4.93 ± 0.98
^a^ HDL-C (mmol/L)	1.26 ± 0.32	1.54 ± 0.36	1.45 ± 0.37^**^
^a^ LDL-C (mmol/L)	2.88 ± 0.80	2.99 ± 0.93	2.95 ± 0.88
^a^ Triglycerides (mmol/L)	1.39 ± 0.83	1.05 ± 0.52	1.16 ± 0.65
Syst-BP (mm HG)	138.07 ± 19.45	132.32 ± 16.98	134.08 ± 17.77
Diast-BP (mm HG)	83.13 ± 9.96	79.18 ± 8.58	80.39 ± 9.11
^a^ Plasma insulin (mmol/L)	57.35 ± 26.68	59.32 ± 55.82	58.67 ± 47.99
^a^ Plasma glucose (mmol/L)	4.65 ± 0.38	4.80 ± 1.21	4.75 ± 1.01
^a^ HOMA status	1.70 ± 0.82	2.11 ± 3.34	1.97 ± 2.77
Functional Disability			
^a^ HAQ	1.74 ± 0.63	1.64 ± 0.56	1.67 ± 0.58

Note: Gender differences indicated as ** = p < .01. Data pertaining to Q-risk-score, ESR, LDL-C, blood pressure and functional disability (HAQ) were available for participants (*N* = (male, female)) as follows; Q-risk-score, *N* = 55 (18, 37), ESR, *N* = 60 (20, 40), LDL-C, *N* = 60 (19, 41) functional disability, *N* = 60 (20, 40). Data for Q-risk variables (e.g., smoking status) are reported for participants who have computed Q-risk score (*N* = 55)

^a﻿^indicates non-normally distributed data that has been log transformed for inclusion parametric statistical tests. *RA* rheumatoid arthritis, *LPA* light physical activity, *MVPA* moderate-to-vigorous physical activity, *Sbreaks* sedentary breaks, *Sbouts* sedentary bouts, *CVD* cardiovascular disease, *Q-risk* Q-risk-score, *CRP* C-reactive protein, *ESR* erythrocyte sedimentation rate, *HDL-C* high-density lipoprotein cholesterol, *LDL-C* low-density lipoprotein cholesterol, *Syst-BP* systolic blood pressure, *Diast-BP* diastolic blood pressure, *HOMA* homeostatic model assessment, *HAQ* Health Assessment Questionnaire

#### Regression analyses

Hierarchical linear regressions were carried out to investigate associations between sedentary time, sedentary behaviour patterns and LPA with 10-year CVD risk. Regression models were first adjusted for accelerometer-wear time and current treatment as necessary (i.e., where significant differences were observed in Q-risk score based on current treatment) [Model 1]. As age and gender were used to compute participants Q-risk score, ﻿regression analyses were not ﻿further-adjusted for these variables (i.e., ﻿avoiding over-adjustment). ﻿Path coefficients (β) for sedentary behaviour variables and LPA were examined to determine the strength and direction of the associations with 10-year CVD risk. Significance was set at *p* < .05. The variance in 10-year CVD risk explained by sedentary time, sedentary bout/break parameters, and LPA engagement were determined via observation of R^2^ values. Where significant associations were observed following initial analyses [i.e., Model 1], models were further adjusted for daily MVPA engagement [Model 2]. Path coefficients were examined to determine whether previously significant associations between sedentary behaviour variables and LPA with 10-year CVD risk were maintained after adjustment for MVPA. Path coefficients representing the relationships between MVPA engagement and 10-year CVD risk were also examined to determine the presence of any significant and independent (of sedentary behaviour patterns and LPA) effects of MVPA on 10-year risk of CVD.

Linear regressions were also conducted to examine associations between sedentary time, sedentary behaviour patterns and LPA with 1) individual CVD risk factors and 2) functional disability. Models exploring these relationships were initially adjusted for age, gender, accelerometer wear-time and current treatment (as appropriate﻿) [Model 1], and further adjusted for MVPA where significant associations were observed [Model 2]. Path coefficients were examined to determine the presence of any significant effects of sedentary behaviour patterns and LPA on individual CVD risk factors and functional disability, independent of MVPA (and vice versa). All analyses were conducted using SPSS.

## Results

Included (*N* = 61) versus excluded (*N* = 36) participants did not differ in terms of age, or any of the targeted outcomes (all *p* > .05). Chi-square tests confirmed the distribution of males to females was not significantly different among excluded compared to included participants (*X* (1) = .27, *p* = .61). No exclusion bias was observed based on ethnicity (*X* (1) = .36, *p* = .55).

Descriptive statistics for included participants’ data are reported in Table 1. Accelerometer data revealed participants spent approximately 8 to 9 h/day sedentary (64.84% of accelerometer wear-time), participated in LPA for 4.5 h/day (32.22% of accelerometer wear-time), and broke up their sedentary time approximately 6 times/h. Interpretation of descriptive data indicated 43% of participants had ≥10% risk of CVD at 10-years based on Qrisk-score. Gender differences were not observed for any sedentary behaviour variables or LPA, but were evident for 10-year risk of CVD (*F* (1, 53) = 10.62, *p* = <.01) and HDL-C (*F* (1, 59) = 9.91, *p* = <.01, Table 1).

At the time of data collection, the number (%) of participants undertaking different pharmaceutical treatments were as follows; Disease Modifying Anti-Rheumatic Drugs (DMARDS, 53%), anti-TNF therapy (10%), Non-Steroidal Anti-Inflammatory Drugs (NSAIDs, 36%), analgesics (33%), cholesterol lowering medication (23%), medication for hypertension (20%). For each type of medication, one-way ANOVAs demonstrated that overall, targeted outcomes were not significantly different on the basis of current treatment. Exceptions were observed for participants taking cholesterol lowering medication (Q-risk score, *F* (1, 53) = 5.88, *p* = .02; M ± SD, yes = 23.54 ± 12.89, no = 13.87 ± 12.87), medication for hypertension (ESR, *F* (1, 58) = 4.49, *p* = .04; M ± SD, yes = 1.23 ± 0.33, no = 0.97 ± 0.39; LDL-C, *F* (1, 58) = 4.13, *p* = <.05; M ± SD, yes = 0.54 ± 0.11, no = 0.60 ± 0.09; and plasma glucose, *F* (1, 59) = 10.41, *p* = <.01; M ± SD, yes = 0.73 ± 0.12, no = 0.66 ± 0.04), and anti-TNF therapy (functional disability, *F* (1, 58) = 5.85, *p* = .02; M ± SD, yes = 1.47 ± 0.14, no = 1.25 ± 0.22). Subsequently, regression analyses which sought to examine associations between sedentary time, sedentary behaviour patterns and LPA with these specific outcomes, were adjusted for current treatment regime as appropriate (see Table 3 legend).

### Correlation analyses

The results of bivariate correlations are displayed in Table 2. This analysis revealed that average daily sedentary time (min/day) and the number of sedentary bouts/day ≥20 min were significantly positively associated with 10-year CVD risk. By contrast, daily LPA (min/day) was significantly negatively associated with 10-year CVD risk. However, the number of sedentary breaks/day and average sedentary bout length were not related to a 10-year CVD risk score.

**Table 2:** Bivariate correlations between accelerometer assessed sedentary behaviour patterns, light physical activity and MVPA with 10-year CVD risk, individual CVD risk factors and functional disability

		1	2	3	4	5	6	7	8	9	10	11	12	13	14	15	16	17	18	19	20
1	Age																				
2	Sedentary time (min/day)	.29*																			
3	^b^LPA (min/day)	−.22	−.78**																		
4	^b^MVPA (min/day)	−.22	−.37**	.35**																	
5	Number of Sbreaks (number/day)	−.12	−.26*	.61**	.03																
6	Number of Sbouts (number/day)	.31*	.81*	−.79**	−.26**	−.67**															
7	Average time per Sbout (min)	.07	.34**	−.50**	−.09	−.56**	.45**														
8	Q-risk	.76**	.37**	−.40**	−.12	−.21	.38**	.20													
9	^b^CRP (mg/L)	.18	.05	−.20	−.24	−.20	.15	.09	.28*												
10	^b^ESR (mm/h)	.12	.20	−.23	−.21	−.09	.21	−.01	.17	.57**											
11	Fibrinogen (g/L)	.25	.20	−.27*	−.23	−.19	.31*	.03	.29*	.69**	.71**										
12	Total cholesterol (mmol/L)	.18	.17	−.10	−.18	.04	.03	.11	.09	.10	.14	− .03									
13	^b^HDL-C (mmol/L)	.05	−.11	.27*	.02	.30*	−.25^†^	−.17	−.33*	−.20	−.10	−.24	.29*								
14	^b^LDL-C (mmol/L)	.15	.22	−.19	−.14	−.13	.15	.19	.19	.22	.19	.06	.86**	−.09							
15	^b^Triglycerides (mmol/L)	.10	.13	−.19	−.17	−.02	.08	.17	.22	−.00	.14	.11	.33*	−.24^b^	.13						
16	Syst-BP (mm HG)	.53**	.19	−.09	−.04	.05	.17	.04	.52**	.16	.19	.17	.11	.28*	−.11	.22					
17	Diast-BP (mm HG)	.37*	.08	.15	.02	.11	.13	.01	.30*	−.05	−.03	−.01	.12	.23	−.07	.22	.71**				
18	^b^Plasma insulin (mmol/L)	−.08	.05	−.11	−.21	−.08	.13	−.08	.16	.28*	.35*	.37*	−.03	−.47**	.08	.37**	.10	.14			
19	^b^Plasma glucose (mmol/L)	.02	.04	−.04	.06	.05	.01	.11	.12	.06	.13	.10	.07	−.30*	.12	.27*	.10	.19	.51**		
20	^b^HOMA status	−.08	.06	−.09	−.17	−.02	.09	−.01	.16	.23	.30*	.31*	−.04	−.47**	.05	.35**	.06	.12	.94**	.74**	
21	HAQ	.09	.13	−.23	−.27*	−.16	.17	.06	.13	.18	.27**	.23	−.04	−.03	.03	−.11	.07	.02	.26**	− .04	.18

Note: * *p* < .05, ** *p* < .01, ^†^ *p* = .05, ^a^ *p* = .06. ^b^ Indicates non-normally distributed data that has been log transformed for inclusion parametric statistical tests

*LPA* light physical activity, *MVPA* moderate-to-vigorous physical activity, *Sbreaks* sedentary breaks, *Sbouts* sedentary bouts, *CVD* cardiovascular disease, *HOMA* homeostatic model assessment, *HDL-C* high-density lipoprotein cholesterol, *LDL-C* low-density lipoprotein cholesterol, *CRP* C-reactive protein, *ESR* erythrocyte sedimentation rate, *Qrisk* Q-risk score, *HAQ* Health Assessment Questionnaire

Sedentary time (min/day) was not related to any individual CVD risk factors. However, the number of sedentary bouts/day ≥20 min was significantly positively associated with fibrinogen. In addition, LPA (min/day) was significantly negatively linked to fibrinogen, and both LPA (min/day) and the number of sedentary breaks/day demonstrated significant positive associations with HDL-C. Average sedentary bout length was not related to any individual CVD risk factors. Daily MVPA (min/day) was significantly and negatively linked to plasma levels of CRP, ESR and fibrinogen.

No significant associations were observed between any accelerometer derived variables with functional disability. However, functional disability was positively correlated with plasma insulin and ESR.

### Regression analysis

#### Model 1

Results from the regression analyses are reported in Table 3. *10-year CVD risk;* the significant positive associations between both daily sedentary time (min/day) and the number of sedentary bouts/day ≥20 min with10-year CVD risk were sustained after adjusting for accelerometer wear-time (sedentary time, R^2^ = .17, number of sedentary bouts ≥20 min, R^2^ = .15). The significant negative association observed between LPA (min/day) and 10-year CVD risk was also maintained in wear-time adjusted models (R^2^ = .18). Finally, the number of sedentary breaks/day, and the average time per sedentary bout ≥20 min remained unrelated to 10-year CVD risk in wear-time adjusted models.

**Table 3:** Regression analyses

	Sedentary time (min/day)				LPA (min/day)				Number of Sbreaks (avg/day)				Number of Sbouts (avg/day)				Average time per Sbout (min)			
	β	R^2^	R^2∆^ ^MVPA^	95% CI [lower, upper]	β	R^2^	R^2∆^ ^MVPA^	95% CI [lower, upper]	β	R^2^	R^2∆^ ^MVPA^	95% CI [lower, upper]	β	R^2^	R^2∆^ ^MVPA^	95% CI [lower, upper]	β	R^2^	R^2∆^ ^MVPA^	95% CI [lower, upper]
Q-risk	.41^**^	.17		.03, .12	−.49^**^	.18		−.14, −.04	−.26	.05		−.54, .04	.39^**^	.15		.90, 4.02	.23	.04		−.29, 2.66
Model 2 ^MVPA^	.44^**^	.17	.00	.04, .15	−.48^**^	.18	.00	−.14, −.04	---	---		--- ---	.38^**^	.15	.00	.80, 4.08	---	---		--- ---
^a^CRP	.06	.00		−.00, .00	−.04	.00		−.00, .00	−.02	.00		−.01, .01	.09	.01		−.03, .07	−.03	.00		−.04, .04
^a^ESR	.25	.05		.00, .00	−.25	.05		−.00, .00	−.20	.02		−.01, .00	.30^*^	.07		.01, .10	−.02	.00		−.04, .04
Model 2 ^MVPA^	---	---		--- ---	---	---		--- ---	---	---		--- ---	.27	.07	.02	−.00, .10	---	---		--- ---
Fibrinogen	.18	.03		−.00, .01	−.18	.03		−.01, .00	−.10	.01		−.03, .02	.26^*^	.06		.00, .27	−.05	.00		−.14, .09
Model 2 ^MVPA^	---	---		--- ---	---	---		--- ---	---	---		--- ---	.22	.09	.03	−.03, .25	---	---		--- ---
Total Cholesterol	.13	.02		−.00, .01	−.13	.01		−.01, .00	.03	.00		−.02, 02	−.01	.00		−.13, .13	.12	.01		−.06, .15
^a^HDL-C	−.17	.03		.00, .00	.18	.02		.00, .00	.16	.02		−.00, .00	−.22	.04		−.01, .00	−.10	.01		−.01, .00
^a^LDL-C	.16	.02		.00, .01	−.17	.02		−.00, .00	.01	.00		−.00, .00	.03	.00		−.01, .01	.13	.02		−.01, .02
^a^Triglycerides	.12	.01		.00, .00	−.09	.01		−.00, .00	.13	.01		−.00, .00	.02	.00		−.01, .02	.13	.02		−.01, .02
Syst-BP	−.02	.00		−.08, .07	−.00	.00		−.08, .08	.12	.01		−.21, .54	−.04	.00		−2.62, 2.01	.01	.00		−2.05, 2.25
Diast-BP	−.13	.01		−.06, .02	.16	.02		−.02, .06	.10	.01		−.13, .27	−.03	.00		−1.38, 1.11	.06	.00		−.90, 1.40
^a^Plasma Insulin	.11	.01		−.00, .00	−.07	.00		−.00, .00	.02	.00		−.01, .01	.15	.02		−.01, .05	−.14	.02		−.04, 01
^a^Plasma glucose	.10	.01		.00, .00	−.12	.01		.00, .00	.13	.01		−.00, .00	.13	.02		−.01, .01	.18	.03		−.00, .01
^a^HOMA	.12	.01		.00, .00	−.09	.01		−.00, .00	.05	.00		−.00, .01	.12	.01		−.01, .04	−.05	.00		−.02, .02
^a^HAQ	.09	.01		−.00, .00	−.11	.01		−.00, .00	−.01	.00		−.01, .01	.07	.00		−.02, .04	−.02	.00		−.03, .02

*Note:* * *p* < .05, ** *p* ≤ .01. ^a^ indicates non-normally distributed data that has been log transformed for inclusion parametric statistical tests

Results are reported as per *Model 1* adjusted for age, gender and accelerometer wear time, and current treatment as appropriate; i.e., Q-risk = cholesterol lowering medication; ESR, LDL-C and fibrinogen = hypertensive medication, functional disability = Anti-TNF therapy). In the case of Q-risk, models are only adjusted for accelerometer wear time and current treatment to prevent over-adjustment (i.e., age and gender are used to calculate composite Q-risk score)

R^2^ represents the variance explained in the dependent variable (10-year CVD risk (Qrisk), individual CVD risk factor or HAQ) by the independent variable of interest (i.e., sedentary time, light physical activity, number of sedentary breaks/day, number of sedentary bouts/day and average time per sedentary bout)

Where initial regression models were significant, daily MVPA was included as a predictor in Model 2. Results are then presented to reflect associations between sedentary behaviour and LPA variables with outcome variables, after adjustment for MVPA (Model 2 ^MVPA^). Model 2^MVPA^ R^2^ represents the variance explained in the dependent variable (Q-risk, individual CVD risk factor or HAQ) by the independent variable of interest after adjustment for MVPA. R^2^ change values (R^2∆^ MVPA), represent the additional variance explained by MVPA for the specific dependent variable of interest (i.e., Q-risk, individual CVD risk factors or HAQ)”

*LPA* light physical activity, *MVPA* moderate-to-vigorous physical activity, *Sbreaks* sedentary breaks, *Sbouts* sedentary bouts, *Qrisk* Q-risk-score, *CRP* C-reactive protein, *ESR* erythrocyte sedimentation rate, *HDL-C* high-density lipoprotein cholesterol, *LDL-C* low-density lipoprotein cholesterol, *Syst-BP* systolic blood pressure, *Diast-BP* diastolic blood pressure, *HOMA* homeostatic model assessment, *HAQ* Health Assessment Questionnaire

##### Individual CVD risk factors

Analyses revealed the previously significant positive associations between both LPA and sedentary breaks with HDL-C (as per bivariate correlations), were attenuated and no longer significant in regression models adjusted for age, gender, accelerometer wear-time and current treatment. However, the number of sedentary bouts/day ≥20 min demonstrated significant positive associations with fibrinogen and ESR in the adjusted models (fibrinogen, R^2^ = .06; ESR, R^2^ = .07). All other associations between sedentary time (min/day), sedentary bout/break parameters and LPA (min/day) with individual CVD risk factors remained non-significant in age, gender, wear-time and treatment adjusted models.

##### Functional disability

The non-significant associations between sedentary behaviour patterns and LPA engagement with functional disability as observed correlation analyses persisted in age, gender and wear-time and treatment adjusted models.

#### Model 2 - Adjustment for MVPA

The significant positive relationships between daily sedentary time (min/day) and the number of sedentary bouts/day ≥20 min with 10-year CVD risk, were maintained following adjustment for MVPA (i.e., these associations were independent of levels of MVPA participation, model R^2^ ^∆ MVPA^, sedentary time = 0.00, sedentary bouts ≥20 min = .00). Similarly, the significant negative association between LPA (min/day) and 10-year CVD risk remained after the inclusion of MVPA in regression models (R^2^ ^∆ MVPA^ = .00). The significant positive association between the number of sedentary bouts/day ≥20 min with fibrinogen and ESR were attenuated and no longer significant following adjustment for MVPA (fibrinogen, R^2∆^ MVPA = .03; ESR, R^2∆^ MVPA = .02).

No significant associations were observed between daily MVPA with 10-year CVD risk, individual CVD risk factors and functional disability (i.e., where regression models were adjusted for age, gender, accelerometer wear-time, and sedentary behaviour or LPA).

## Discussion

This study is the first to examine the associations between objectively assessed daily sedentary time (≤1.5 METS), sedentary behaviour patterns, and LPA engagement (1.6 – 2.9 METS) with long-term (10-year) CVD risk in RA. Results revealed daily sedentary time and the number of sedentary bouts/day ≥ 20 min were positively associated with 10-year CVD risk, with the reverse relationship evidenced for daily LPA participation. Importantly, these significant relationships were observed to be independent of levels of daily MVPA engagement.

Our results suggest that daily sedentary time and LPA engagement may hold implications for 10-year risk of CVD in RA. These findings are aligned with those reported in population based studies, which demonstrate the role of sedentary behaviour and LPA for long-term risk of CVD among adults (e.g., risk of a first atherosclerotic cardiovascular disease event) [58, 59]. To illustrate the clinical significance of current findings, interpretation of path coefficients indicate that reducing sedentary time by 68 min/day (i.e., the standard deviation), would equate to a 5.5% reduction in 10-year CVD risk, regardless of an individual’s level of MVPA engagement. Similarly, increasing LPA by the same amount would correspond to a 6% reduction in 10-year risk of CVD. Of relevance to these findings is the high correlation between LPA and sedentary time that is also observed in this study. This is indicative of a ‘displacement association’ whereby the action of reducing sedentary time is likely to be synonymous with increasing LPA among people with RA [60]. As such, any improvements in long-term CVD risk derived from increasing participation in LPA, are likely to be equivalent to those resulting from associated reductions in sedentary time among this patient group. Taken together, our results provide the first evidence to suggest that reducing sedentary time engagement and increasing participation in LPA (i.e., “sit less, move more”), may help to attenuate long-term CVD risk in RA.

The point that current associations between sedentary time and LPA with 10-year risk of CVD, were observed independently of levels of MVPA participation is particularly pertinent to this contention. Indeed, due to the pain symptomatic of RA and concomitant restricted mobility, people with RA represent a patient group for whom engagement MVPA may be challenging. Consequently, it is likely that for these individuals, reducing sedentary time and increasing engagement in LPA, will be perceived as more feasible than realising higher levels of MVPA participation.

We show for the first time that the number of sedentary bouts/day ≥20 min is positively associated with 10-year CVD risk among RA patients. As such, restricting sedentary bouts to less than 20 min via interrupting sedentary time with LPA, may be of benefit towards reducing long-term CVD risk for this patient group. This may also represent a palatable health promotion message for people with RA, e.g., aim to break up your sedentary time every 20 min with LPA. Still, emphasis should also be placed on the *duration* of the interruption in sedentary time for this population, not just the act of interrupting itself (i.e., a sedentary break). Indeed, no association was observed between the number of sedentary breaks/day and 10-year risk of CVD in this study. Thus, the simple act of interrupting sedentary time may not be sufficient to induce the necessary physiological mechanisms (e.g., improvements in lipid profile) in order to improve long-term CVD risk for RA patients. Further research is therefore warranted to infer the optimal ‘sedentary break’ duration and associated subsequent intensity of PA engagement likely to induce the required physiological signal to contribute towards a more favourable long-term CVD-risk profile in RA. For example, a recent study revealed that interrupting sedentary time with 2 min periods of light intensity activity was associated with lower systolic blood pressure in overweight and obese adults [61].

Considering that calculated cardiovascular risk score is a composite score, this raises the question of specifically which *modifiable* factors may contribute towards the significant relationships observed herein. Secondary aims of this study were to explore associations between sedentary behaviour patterns and LPA with individual CVD risk factors, thus enabling the contribution of specific factors to be examined. Our results revealed that both daily sedentary time and LPA demonstrated small to moderate non-significant relationships with total and HDL-cholesterol in adjusted analyses (β = −.13 to .18). Whilst not significant, the size of these associations is comparable to significant associations observed in a previous study of RA, in which associations between accelerometer assessed sedentary behaviour and LPA with individual CVD risk factors were examined [62]. Future research should therefore aim to explore the possibility that variability in cholesterol levels as related to levels of sedentary behaviour and LPA, may represent a physiological pathway through which these behaviours are associated with long-term risk of CVD. This proposition is aligned with the hypothesis that down-regulation of lipoprotein lipase (LPL) activity - an enzyme which catalyses the hydrolysis of circulating triglycerides – is a key physiological mechanism underlying the adverse association between sedentary behaviour and CVD risk. Specifically, LPL activity decreases in response to sedentary behaviour, potentially holding adverse implications for cholesterol profile (e.g., lower HDL-cholesterol) [63].

With regards to examination of the relationships between sedentary time, sedentary behaviour patterns and LPA with 1) individual CVD risk factors and 2) functional disability, the lack of significant associations reported herein are inconsistent with findings from extant research [22–24, 36–40, 43, 46, 47, 64]. Only the number of sedentary bouts/day  ≥ 20 min was significantly related to secondary outcomes (i.e., positive associations with fibrinogen and ESR). Previous studies conducted among non-RA populations, have revealed sedentary time, sedentary behaviour patterns, and LPA to be linked to serological markers of CVD and cardio-metabolic health (e.g., CRP, HDL-cholesterol, blood pressure and plasma glucose, HOMA status), as well as physical function in both healthy adults and clinical cohorts. Our findings also contradict results reported by Khoja et al., (2016) - the only existing study to examine associations between objectively assessed sedentary behaviour and LPA with markers of CVD and physical function in RA specifically [62]. In the case of Khoja and colleagues (2016), incongruent findings may represent inconsistencies with regards to the way sedentary behaviour and LPA were defined. In this study, we applied the widely accepted ≤1.5 MET definition of sedentary behaviour, as advocated by the sedentary behaviour research network [35], and considered LPA to represent activities requiring 1.6 – 2.9 METs [65, 66]. In contrast, Khoja et al., (2016) defined sedentary behaviour and LPA as activities requiring <1 MET, and 1 – 2.9 METS, respectively. As a consequence, this may have resulted in exclusion of common sedentary behaviours requiring energy expenditure of 1 – 1.5 METS (e.g., sitting and reading a book or typing [65, 67]), and subsequent underestimation of sedentary time engagement/overestimation of LPA.

Still, current results diverge from those observed among such studies of non-RA populations, in which sedentary behaviour (≤1.5 METs) and LPA (1.6–2.9 METS) are reported to be related to CVD risk profile and physical function [22–24, 36–40, 43, 46, 47, 64]. The absence of significant relationships in the current study, may therefore be due in part to the disproportionately higher levels of inflammation observed in RA, relative to other populations. That is, the effects of sedentary behaviour and LPA on specific serological markers of CVD and physical function, may be comparatively small when considering the chronic and elevated-inflammatory disease-state characteristic of RA. Thus, in order to reduce systemic inflammation, and improve physical function in RA, the dose and intensity of PA engagement may need to be higher than reported for non-RA populations (i.e., MVPA vs LPA, respectively).

Limitations to the current study include a restricted sample size following data reduction procedures, and a cross-sectional study design. As outlined, longitudinal and experimental studies among larger samples are therefore required to confirm the relationships reported herein. Still, this is the first study to employ accelerometers to explore the implications of sedentary behaviour patterning and LPA for cardiovascular health among RA patients. We also acknowledge that conducting repeated regression analyses to examine the hypothesised associations might have inflated the chance of Type 1 error. However, this is the first study to examine relationships between sedentary behaviour patterns and LPA across a broad range of CVD related outcomes in RA. Therefore, we believe that it is of interest to analyse and report results pertaining to all relevant CVD variables examined within the PARA study, even in the instance that null or significant relationships were observed. Finally, the use of the GT3X in this study means the definition of sedentary behaviour employed considers only energy expenditure (i.e., ≤1.5 METs). That is, we were not able to examine whether behaviours characterised by ≤1.5 METS (<100 counts per minute), occurred whilst sitting/lying vs. standing. Still, studies to date examining the implications of sedentary behaviour for health across diverse populations have largely employed accelerometers to measure sedentary time [52]. Thus, the results presented herein facilitate comparisons with other relevant literature. Nevertheless, future studies should seek to employ devices that enable assessment of posture (e.g., the activPAL) alongside traditional accelerometry based approaches used to measure sedentary time, in order to more accurately determine the implications of sedentary behaviour for health outcomes in RA.

## Conclusion

In conclusion, findings suggest that decreasing sedentary time and increasing participation in LPA may contribute towards reduced 10-year risk of CVD amongst patients with RA, independently of levels of MVPA engagement. In particular, limiting sedentary periods to bouts ≤20 min in length, may attenuate 10-year CVD risk for this patient group. Intervention and experimental studies which examine the specific mechanisms responsible for contributing towards a more favourable CVD risk profile are necessary to extend these findings.

## Abbreviations

ANOVA: Analysis of variance
CRP: C-reactive protein
CVD: Cardiovascular disease
ESR: Erythrocyte sedimentation rate
HAQ: Health assessment Questionnaire
HDL-C: High-density lipoprotein cholesterol
HOMA: Homeostasis models assessment
LDL-C: low-density lipoprotein cholesterol
LPA: Light physical activity
LPL: Lipoprotein Lipase
METS: Metabolic equivalents
MVPA: Moderate-to-vigorous physical activity
PA: Physical activity
RA: Rheumatoid Arthritis
